# Closed-loop modulation of remote hippocampal representations with neurofeedback

**DOI:** 10.1016/j.neuron.2024.12.023

**Published:** 2025-01-20

**Authors:** Michael E. Coulter, Anna K. Gillespie, Joshua Chu, Eric L. Denovellis, Trevor T.K. Nguyen, Daniel F. Liu, Katherine Wadhwani, Baibhav Sharma, Kevin Wang, Xinyi Deng, Uri T. Eden, Caleb Kemere, Loren M. Frank

**Affiliations:** 1 Kavli Institute and Department of Physiology UCSF; 2 Howard Hughes Medical Institute, University of Washington; 3 Departments of Biological Structure and Lab Medicine and Pathology, University of Washington; 4 Neuroengineering Initiative, Rice University; 5 SpikeGadgets Inc.; 6 Dept. of Statistics, Beijing University of Technology; 7 Dept. of Mathematics and Statistics, Boston University

**Keywords:** memory retrieval, hippocampus, neurofeedback, place cells, spatial decoding

## Abstract

Humans can remember specific remote events without acting on them and influence which memories are retrieved based on internal goals. However, animal models typically present sensory cues to trigger memory retrieval and then assess retrieval based on action. Thus, it is difficult to determine whether measured neural activity patterns relate to the cue(s), the memory, or the behavior. We therefore asked whether retrieval-related neural activity could be generated in animals without cues or a behavioral report. We focused on hippocampal “place cells” which primarily represent the animal’s current location (local representations) but can also represent locations away from the animal (remote representations). We developed a neurofeedback system to reward expression of remote representations and found that rats could learn to generate specific spatial representations that often jumped directly to the experimenter-defined target location. Thus, animals can deliberately engage remote representations, enabling direct study of retrieval-related activity in the brain.

## General significance

Animal models of memory retrieval trigger retrieval with cues and measure retrieval using behavior. To isolate neural activity associated with retrieval, Coulter et al. developed a neurofeedback paradigm that rewards hippocampal activity patterns associated with retrieval. Rats learned to generate these patterns. This paradigm enables direct study of retrieval-associated activity.

## Graphical Abstract

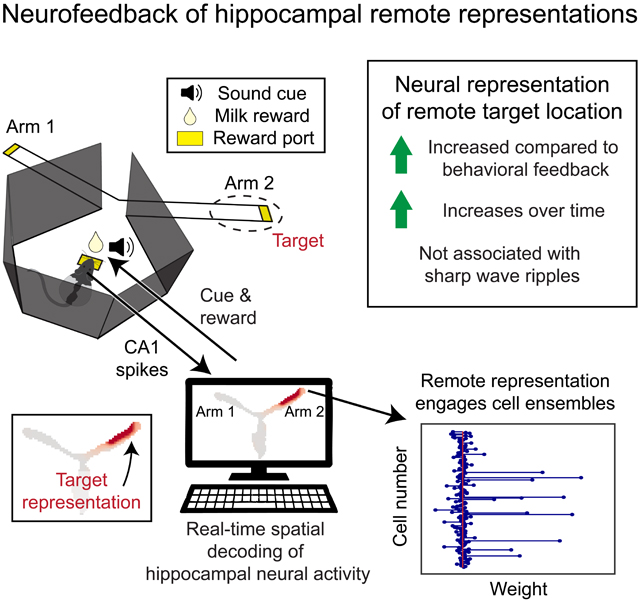

## INTRODUCTION

Remembering is distinct from acting. Humans can remember places we have been and experiences we have had without overt behavioral signs that these memories have been retrieved. Remembering can also happen in the absence of specific external retrieval cues. Humans can retrieve specific memories based on internal goals^1^, and in some cases memories can even seem to be retrieved spontaneously^2^. Finally, remembering an experience distant in place or time does not seem to require mentally traversing all intermediate places or times; instead the brain is able to mentally teleport or “jump” directly to the memory^3^. Thus, memory retrieval is a process that can be expressed in the brain separately from: (1) the decision to act based on the content of the memory, (2) specific external cues that trigger the memory, and (3) intervening experiences that separate the current state from the past event.

In current animal models and associated experimental paradigms, memory retrieval is not well isolated from these co-occurring processes, limiting our ability to understand the activity patterns that support retrieval. First, current approaches typically assess whether a memory has been retrieved based on a behavioral report. Widely used paradigms including contextual-fear conditioning^4–6^, the Morris Water Maze^7^, and many others, place animals in a familiar context and quantify memory based on whether the animals exhibit specific behaviors (e.g., freezing or directed navigation to a remembered goal location). Activity patterns measured in these paradigms are the result of a complex combination of sensory information processing, memory retrieval, a decision process, and action, and so it is difficult to isolate and study the retrieval process separate from other ongoing computations.

Second, current experimental approaches typically engage retrieval by presenting external cues such as those found in a particular spatial context^7–10^. These cues can strongly influence patterns of brain activity, including spiking in brain regions like the hippocampus^11^ that contribute to memory formation and retrieval^9^. Thus, representations of the current sensory inputs can be difficult to disentangle from memory-related activity.

Third, current paradigms do not require that animals retrieve internal representations associated with experiences distant in time and place. Even in cases where animals must navigate to a distant location^7^, or where behavior depends on a past trial^12^, we cannot be sure that any particular remote memory is being retrieved. Instead, animals may use alternative strategies where they construct movement vectors toward a goal or makes choices based on familiarity.

How, then, can we directly study memory retrieval in animals? Brain-machine interface (BMI) paradigms provide a powerful approach that has the potential to enable these studies. Numerous experiments have shown that subjects can learn to control a specific neural activity pattern (from single neuron spiking to population-level activity) via continuous visual or auditory feedback (e.g., moving a cursor on a screen to a goal location)^13–17^. As a result of the sensory feedback, the subject learns to incrementally change the target neural activity pattern to reach a goal state. In the context of the hippocampus, a BMI device using this approach was recently developed wherein rats learned to mentally navigate through a virtual reality environment^18^. The device updated the visual display corresponding to a location in the environment based on population activity in hippocampus and thus, provided continuous visual feedback to the animal. In this environment, rats learned to generate representations of continuous spatial trajectories from the current position to a goal location.

These previous results demonstrate that with continuous sensory input, the brain can learn to generate the next correct pattern in a sequence to achieve a goal. However, two additional features are required to enable direct study of retrieval of a spatial memory. First, the retrieval-related pattern would need to be generated in the absence of the specific (confounding) sensory input associated with the location. Second, the retrieval related pattern would need to be generated without requiring the generation of the full intervening sequence of places between the current position and the remembered location.

We therefore developed a real-time neurofeedback paradigm that did not present memory-specific cues and did not require traversal of a complete mental trajectory to arrive at a retrieved location. In our paradigm, rats were rewarded for generating remote hippocampal spatial representations. We focused on hippocampal activity patterns as a substrate for retrieval, both because the hippocampus is critical for spatial and event memory retrieval for recent experiences^9^ and because hippocampal activity patterns permit the experimental identification of activity consistent with retrieval. Specifically, as an animal explores an environment, each hippocampal place cell is predominately active when the animal occupies one or more locations in an environment (the cell’s “place fields”). Sets of place cells can also “reactivate” when the animal is far outside the cells’ place fields^11,19–25^, reinstating a remote representation. These events can also include representational jumps to remote locations that would not be possible during real movement^26^, and these events are associated with activity across the brain consistent with the retrieval of a memory^24,27,28^. Further, artificial activation of a remote representation^29^ or association of the representation with reward can be sufficient to drive associated behaviors^29,30^.

Using this paradigm, we found that rats could learn to generate specific remote hippocampal spatial representations in the absence of sensory cues indicating which representations to generate. Strikingly, in most cases, these representations jumped to the target region without representing the intermediate locations between the animal and the target. Further, these representations were largely expressed in a brain state not previously associated with remote spatial activity patterns. This work establishes a model for studying spatial memory retrieval in the absence of sensory cues and specific behavioral outputs.

## RESULTS

### Closed-loop hippocampal neurofeedback

**Figure 1.**
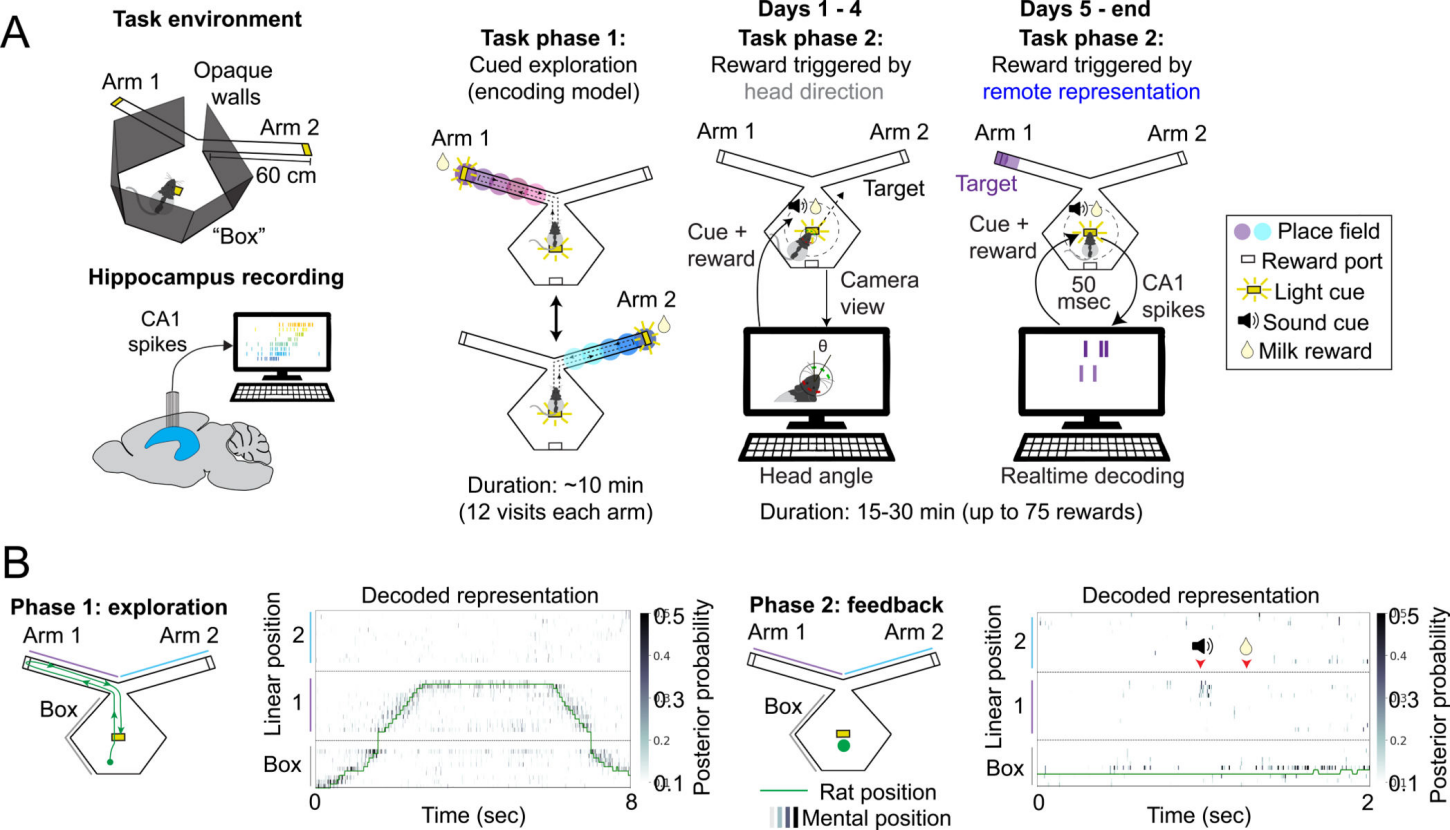
Closed-loop hippocampal feedback system. **(A)** The task environment consisted of a central “box” area with a central reward port and two arms, each with a reward port at the end. The end of one of the two arms was used as the target location for neurofeedback in each session. Note that the walls surrounding the central box are opaque. Each task session contained two task phases, exploration and feedback. During the feedback phase, either specific head directions or remote target representation were detected and triggered a tone. A nosepoke at the center well within 3 seconds of the tone then triggered delivery of reward. **(B)** Clusterless decoding of hippocampal activity accurately tracked the rat’s actual position during movement. During feedback, the decoder detected remote representations that triggered tone and reward.

We developed a behavioral task and a real-time neurofeedback system that rewarded animals for generating specific, experimenter-chosen remote representations. Six rats were surgically implanted with tetrodes targeting hippocampus and following recovery, participated in a feedback-based task in a modified Y-maze equipped with lighted reward ports (Fig. 1a, S1a, S2a). In each of three daily sessions, rats were first visually cued to explore the left and right arms of the environment (Fig. 1a, Task phase 1). Following this exploration period, the lighted ports in the outer arms were extinguished, and the feedback portion of the session began at a central reward port away from the arms (Fig. 1a, Task phase 2).

The feedback portion of the task was divided into two stages. In both stages animals were required to remain near the center well of the track to receive reward. During the first stage (days 1–4 of training), rats received behavioral feedback based on head direction. If the rat turned its head in the specified direction (either left or right) and was near the central reward port, a reward-cue tone played. If the rat then nose-poked in the port within 3 sec, reward was delivered (see Methods).

Following the four days of head direction feedback, we switched to the second, neurofeedback stage. In this stage animals were rewarded only if they generated a hippocampal representation of a remote target region: the distal end of either the left or right arm of the track (see Methods and Fig. 1a). This remote representation was identified by a real-time system that continuously decoded (every 6 milliseconds) the hippocampal representation of space using an encoding model that related hippocampal spiking activity to the rat’s position during the exploration phase of each session (Fig. S1a-e)^26,31,32^. If the decoder detected a temporally extended representation of the target region, the tone was presented. The animal was then rewarded if it nose-poked in the central well within 3 seconds (Fig. 1a). The target region was held fixed for three days (nine sessions) and then switched to the other arm. This switching continued for 6–18 days per animal depending on the quality of the recordings. In each session, the feedback period lasted for either 30 minutes or until animals received 75 rewards. Importantly, during the feedback period, the animal could not see the target location at the arm end from central port, and no target-location-specific sensory cues were presented.

**Figure 2.**
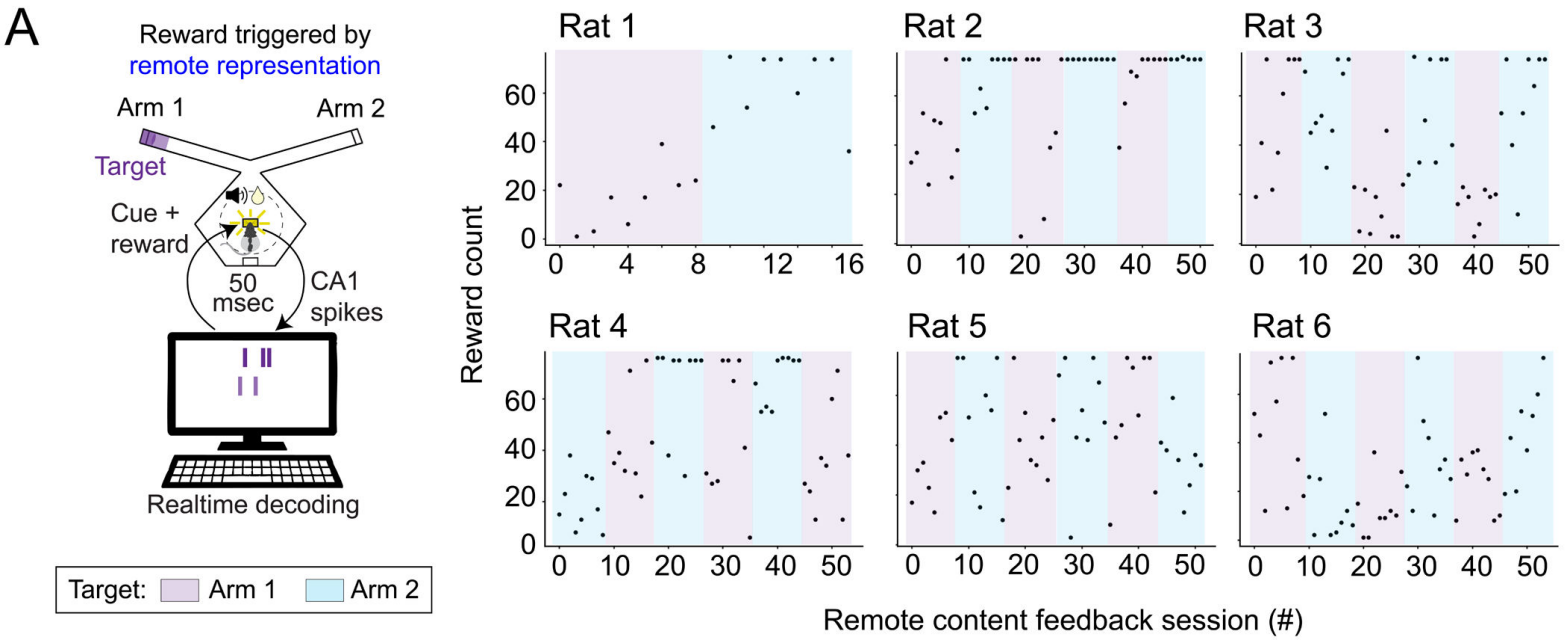
Rewards received during neurofeedback task. **(A)** Each rat maximized rewards during some neurofeedback sessions. Maximum of 75 rewards per session.

Behavioral results showed that all six rats were able to rapidly and consistently maximize the number of rewards per session with behavioral feedback based on head direction (Fig. S2d). Further, all six rats also reached the maximal number of rewards per session during remote representation neurofeedback, although there was substantial variability across sessions and rats; some animals achieved high performance in many sessions while others achieved high performance in a smaller fraction of sessions (Fig. 2a).

To analyze the data from the neurofeedback sessions, we first validated the accuracy of our real-time decoder in post-experimental analyses where we used the encoding model to decode the actual location of the animal during the cued exploration period (Task phase 1; Fig. 1b, left; Fig. S2b,c; Methods); a small number of lower quality decoding sessions were excluded from further analyses. We then examined the representations that triggered reward in the neurofeedback sessions to validate the real-time system. As expected, the spatial content was specific to the chosen remote target, while during head direction feedback sessions, the rat’s current location was typically represented (Fig. 1b, right, S3a,b,c).

**Figure 3.**
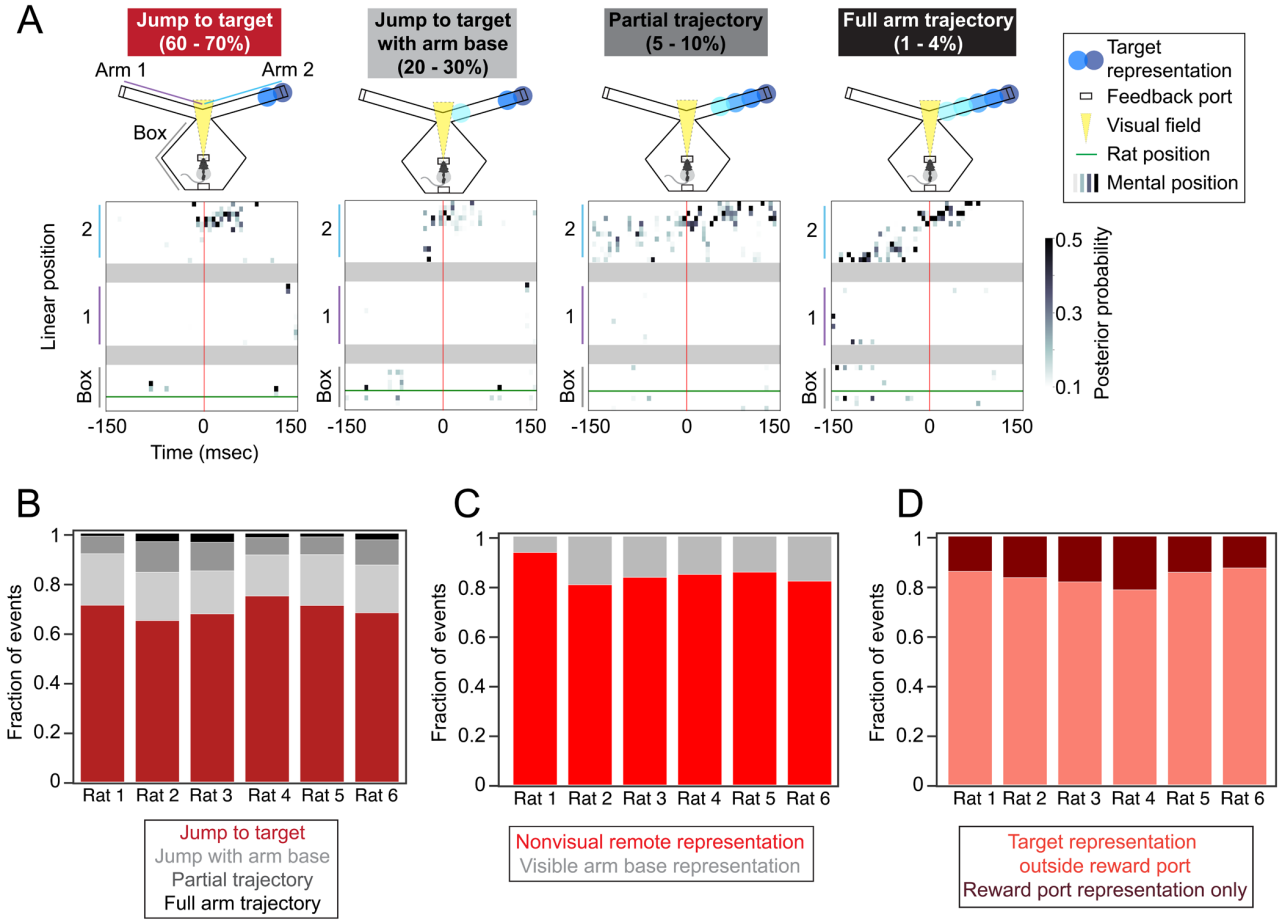
Remote representations jump to target location. **(A)** Individual detected remote representations. Representation classification and frequency are noted above each example. Categories: jump (posterior mass >0.4 in 25 cm at the end of the arm), jump and arm base (posterior mass >0.2 in 15 cm at the base of the arm), medium trajectory (representation with significant linear regression covering at least 35 cm in target arm), long trajectory (representation with significant linear regression covering at least 45 cm in target arm). Colored circles on track indicate decoded location of spatial representation. **(B)** Summary of event classification for all rats. This includes 90 msec before detection. **(C)** Fraction of detected events with representation of the arm base within the visual field (first 5 cm of arm). **(D)** Fraction of detected events with representation of the exact reward port location (last 5 cm of arm).

We found that animals solved this task by generating representations that often jumped to the target region, consistent with mental teleportation, a key capacity of memory retrieval. Inspection of individual representations (up to 90 msec before detection) showed that most were confined to the target region, some included the target region and the start of the target arm, and rarely, they included partial and full remote sequences towards the target (Fig. 3a). Across all rats, 65–75% of detected representations included only the target location or jumped from the current location to the target, while only 1–4% of representations were sequential trajectories down the length of the arm (Fig. 3b). Most representations (60–78%) jumped at least 50 cm from the rat’s current location (Fig. S4a).

Our results also suggest that neither specific sensory cues nor reward-specific representations contributed substantially to the observed remote activity patterns. First, when the animals were close to the center well as was required for reward to be delivered, they were able to see only the very beginnings of the outer arms. If this sensory input triggered retrieval, we would expect that the resulting representation would include the beginning of the arm associated with the target location. This was not the case: these representations were present in 7–19% of events across rats (Fig. 3c), suggesting any available cues were only contributing to a small fraction of events. Second, although the target location included the reward port at the very end of the arm, detected remote representations only occasionally included a representation of the reward port location (13–22% across rats; Fig. 3d), showing that generation of remote representations was not primarily driven by the target being a reward location.

### Increased representation of the target location

**Figure 4.**
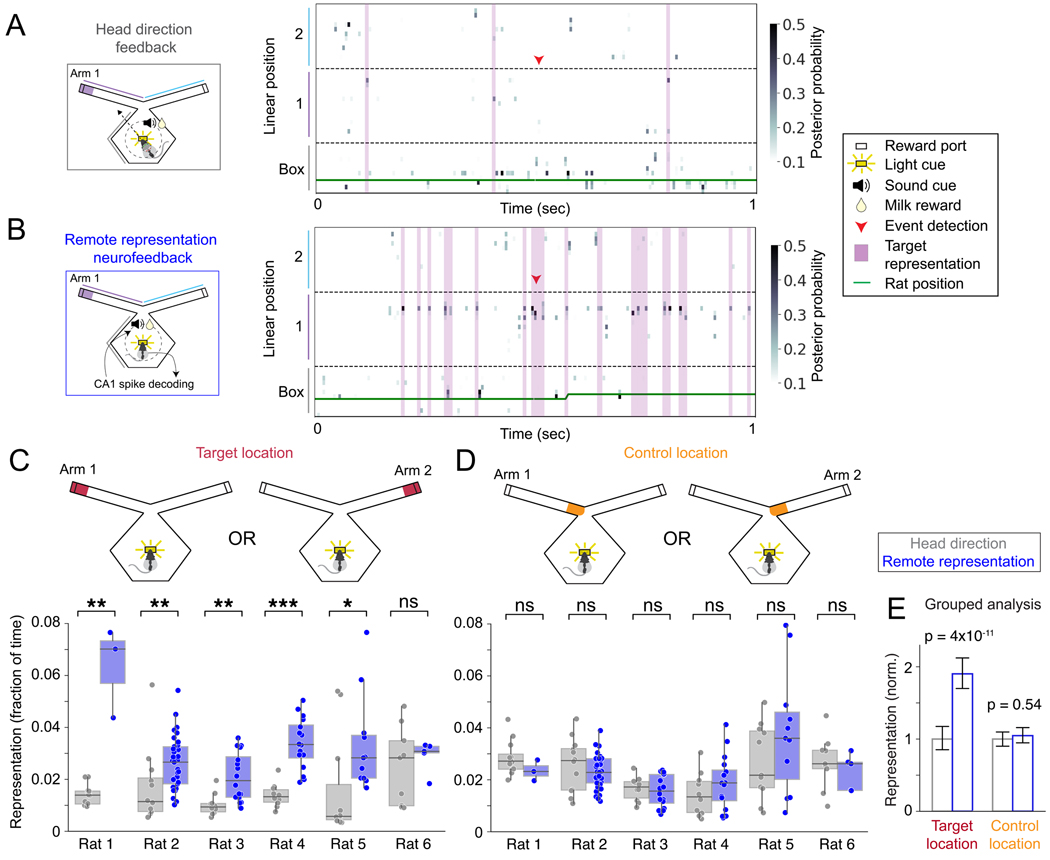
Increased hippocampal remote representations during neurofeedback. **(A)** Head direction feedback (schematic at left) and example of decoded mental position around the time of a detected correct head direction event (right). **(B)** Remote representation neurofeedback session (schematic at left) and example of decoded mental position around the time of a remote representation detection event (right). Spatial representation was calculated by counting the number of 6 msec time bins (purple shading) while the rat was near the center reward port (within 17 cm) and >40% of the decoded mental position (posterior mass) matched the specified location. To match the amount of reward for head direction and remote representation conditioning, only sessions when the rats received >90% of the maximum rewards were included. **(C)** Prevalence of target location representation (25 cm at end of target arm) during high-reward sessions for head direction feedback vs. remote representation feedback sessions for each rat. **(D)** Prevalence of control location representation (non-rewarded, 25 cm at base of target arm) during high-reward sessions for head direction feedback vs. remote representation feedback sessions for each rat. **(E)** Grouped analysis across all 6 rats: LME (test variable is session type). Mann-Whitney test (individual rats), *: p<0.05, **: p<0.01, ***: p<0.001. *n*: see Table S1. Box plots: center line is median, box is inner quartiles, whiskers are full distribution except outliers.

Our system established relatively strict criteria for a remote spatial representation (see Methods), and a detailed inspection of the decoded representations present during the neurofeedback periods revealed numerous instances of apparent target representations that did not meet these criteria. Therefore, to determine whether target representation was consistently enriched through neurofeedback (which would provide further evidence that animals can control representation content), we calculated the fraction of time that at least 40% of the representation (probability mass) was within the target region while the rat was at the center port (see Methods; Fig. 4a,b). During these periods, the majority of the representation was almost always within the target arm (Fig. S4b).

We then compared remote representation during head direction feedback sessions to remote representation during neurofeedback sessions. Because reward itself can drive patterns of hippocampal activity that express remote representations^33,34^, we restricted our analyses to sessions with similar reward amounts (>90% of possible rewards). We found that remote representation neurofeedback drove a substantial increase in the generation of representations of the target region as compared to the head direction feedback. Representations of the target region were approximately two-fold more prevalent during neurofeedback sessions compared to head direction feedback sessions (Fig. 4c,e, individually significant in 5 of 6 rats (p < 0.05, Mann-Whitney); grouped analysis (linear mixed effects model, LME): all data: p = 3.9e-11, tone-triggering representation removed: p = 3.6e-11; Fig. S5a). Importantly, this did not reflect a non-specific increase in remote representations: representations of a physically closer location (the base of the target arm) were not more prevalent in neurofeedback sessions compared to head direction feedback sessions (Fig. 4d,e, non-significant in each rat, grouped analysis: p = 0.54, LME). Likewise, representation of the end of the opposite (non-target) arm was not enriched (Fig. S5c, grouped analysis: p = 0.067, LME). We also examined the relationship between task performance (number of rewards received) and the amount of target representation and found that these measures were significantly positively correlated in five of the six animals (p < 0.01 for rats 1, 2, 3, 5 and 6) and weakly negatively correlated in one animal (p = 0.022, rat 4).

**Figure 5.**
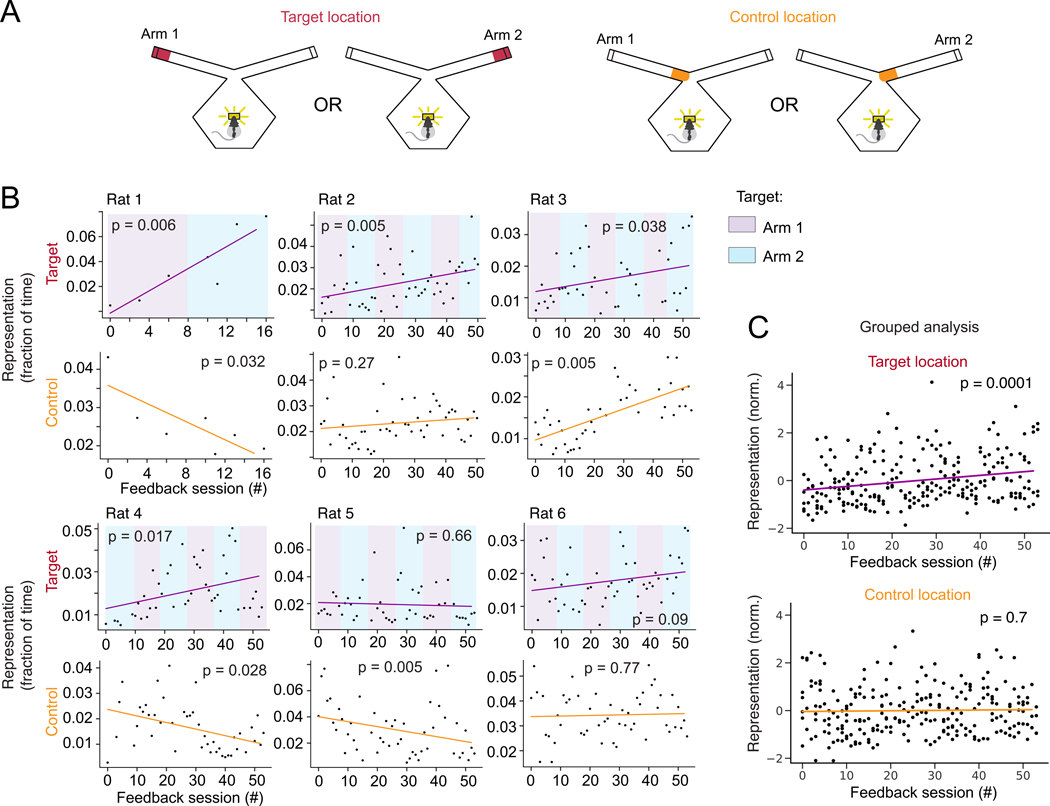
Remote hippocampal representations increase over time with neurofeedback. **(A)** Target location is the arm end and control location is the arm base. **(B)** Target location representation prevalence across all remote representation feedback sessions (red) and for control location representations (orange). Colors in top plots represent designated target arm. Line shows linear regression fit; p-value corresponds to the slope of the linear fit. **(C)** Grouped analysis (linear regression) for normalized data (z-scored) from all 6 rats for target (top) and control locations (bottom). *n*: see Table S1.

The prevalence of these target representations increased with time, consistent with a learning process. We examined target representation across all included remote representation neurofeedback sessions (6 or 18 days) and found that even though the target representation switched every three days, the overall prevalence of the target representation increased over time (Fig. 5, individually significant in 4 of 6 animals; grouped analysis: linear regression of z-scored values, all data: p = 0.0001, tone-triggering representation removed: p = 0.0002; Fig. S5b). By contrast, there was no consistent change in the representation of the base of the target arm (Fig. 5, significant increase in only one animal; grouped analysis: linear regression of z-scored values, p =0.7). These findings complement the analyses of content for the reward triggering events (Fig. 3b) and confirm that rats most often generated representations with discontinuous jumps from the animal’s current location to the distant target location rather than representations that moved sequentially to the target location. Finally, we also noted that representation of the opposite arm end also increased over time (Fig. S5d, linear regression of z-scored values, p = 0.004), perhaps because the target switched back and forth between the two arms every three days. Overall, the longitudinal increases in representation were specific to rewarded locations.

### Remote representations engage hippocampal cell assemblies

The real-time system and the associated analyses presented above used all CA1 spikes above an amplitude threshold to assess the structure of hippocampal spatial representations. This approach provides more accurate decoding than using only spikes that can be confidently associated with single neurons^35,36,37^, but limits the conclusions that can be reached regarding single neuron activity. As memory retrieval is thought to engage the coordinated activity of ensembles of neurons^38^, we also performed spike sorting and analyzed the resulting putative single neuron data to determine whether and how ensembles were engaged during periods of remote representation.

**Figure 6.**
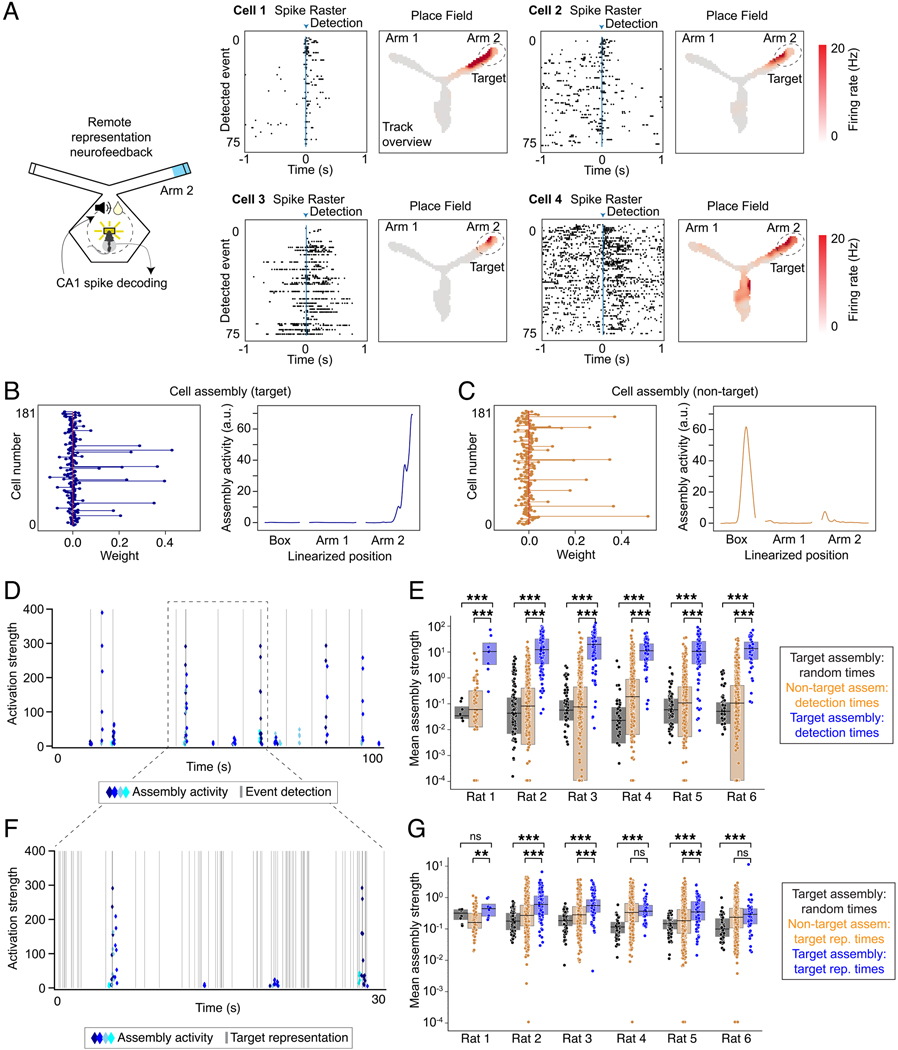
Cell assemblies are activated at the time of remote representations. **(A)** Individual neurons active at remote representation detection times. For each cell, left: raster plot showing spikes surrounding each detected remote representation (blue arrow); right: place field computed during exploration phase (2D occupancy normalized firing rate, 3 cm^2^ bins). **(B)** Target cell assembly. Left: individual cell weights; right: combined location-specific assembly activity. **(C)** Same plot as **(B)** for a non-target cell assembly. **(D)** Example of activation strength for four target assemblies at the time of remote representation detection. Blue diamonds show activity of each target assembly and grey lines indicate detection times. **(E)** Target assembly activity at time of remote representation detection (blue) compared to random times (black) and to non-target assemblies at detection times (orange) across all sessions. Assemblies with maximum strength > 100. **(F)** Zoom-in on plot from **(D)** showing target representation times (grey lines) and assembly activity (blue diamonds). **(G)** Target assembly activity at time of remote representation (>40% posterior mass in target location) outside of detection events (blue) compared to random times (black) and to non-target assemblies at remote representation times (orange) across all sessions. In **(E)** and **(G)**, for plotting only, any values less than 1e-4 were set to 1e-4. Mann-Whitney test, **: p < 0.01, ***: p < 0.001. *n*: see Table S1. Box plots: center line is median, box is inner quartiles, whiskers are full distribution except outliers.

Our results demonstrate engagement of coordinated ensembles of single neurons during remote spatial representations. We often found that multiple single neurons had place fields (increased firing rate) in the target region and that these individual neurons were active around the time of target representation detection (Fig. 6a). These included neurons that showed a pronounced increase in activity at detection of the reward-triggering representation (cells 1 and 2) and others that were active before, during, and after the detection of the reward-triggering representation (cells 3 and 4). We quantified this engagement by identifying coordinated activity of groups of neurons (cell assemblies^39^; see Methods) and segregating them into two groups based on whether or not their activity represented the target location during the exploration period of each session (target and non-target assemblies; Fig. 6b,c). We then computed the activity of these assemblies at the times when remote representations were expressed.

We first focused on times when the target representation was detected (and the reward cue was triggered) during the feedback period of the neurofeedback sessions. During these periods, when the animal was in the box, far from the actual target location, we nevertheless found strong and specific activation of target-representing assemblies: these assemblies were about 89 times more active at detection times compared to random times (Fig. 6d,e, each rat p<0.001, Mann-Whitney test) and were about 26 times more active than non-target assemblies (Fig. 6d,e, each rat p<0.001, Mann-Whitney test). In addition, animals frequently generated representations of the target location outside the times of rewarded events. During these periods, target assemblies were about 3.9 times more active than at random times (Fig. 6f,g, 5 of 6 rats p<0.001, 1 rat, p=0.16, Mann-Whitney test; grouped analysis of all rats, p = 3.2e-27, LME) and were about 1.7 times more active than non-target assemblies at these same times (Fig. 6g, 3 of 6 rats, p<0.001, 1 rat: p=0.005, 2 rats: p=0.4, 0.45, Mann-Whitney test; grouped analysis, p = 9.5e-18, LME). We note that target assembly activity was not detected during all times of remote representation; this is likely because our system used clusterless decoding (decoding without cell clustering using all spikes above a voltage threshold), which incorporates many spikes that are not clustered into single units during spike sorting^40^.

We also verified that detection events most often engaged multiple neurons (illustrated in an example, Fig. S6a) by counting the number of high-strength cells in target assemblies that were active before each target detection event (Fig. S6b, Methods). Across all sessions, rats had an average of 1.8 – 8.1 cells active immediately preceding each detection event, which corresponded to 10 – 14% of high assembly strength cells (Fig. S6c). This is comparable to the number of neurons expressing remote representations during movement periods as reported in previous papers^22,23^, and given that we are recording a tiny fraction of the total number of neurons in the hippocampus, suggests that many neurons are engaged during each of these remote representation events. Finally, two additional analyses examining cosine distances and explained variances indicated that spiking patterns during remote representation times were more similar to patterns seen during actual exploration than to patterns seen at random times (Fig. S6e,f)

We then asked whether there were consistent changes in spatial representations associated with the generation of remote representation. Previous work has shown that the firing fields of place cells can move (remap) within the same environment when the location of reward changes^41^. Because rats in this task received reward at the center port while representing a remote location, and because the animals were activating representations of a remote location, it could have been the case that there were consistent reallocations of activity from the remote location to the center. This was not consistently true across animals, but there was some evidence for remapping in two of the six animals (Fig. S6d).

### Brain state during remote representation events

**Figure 7.**
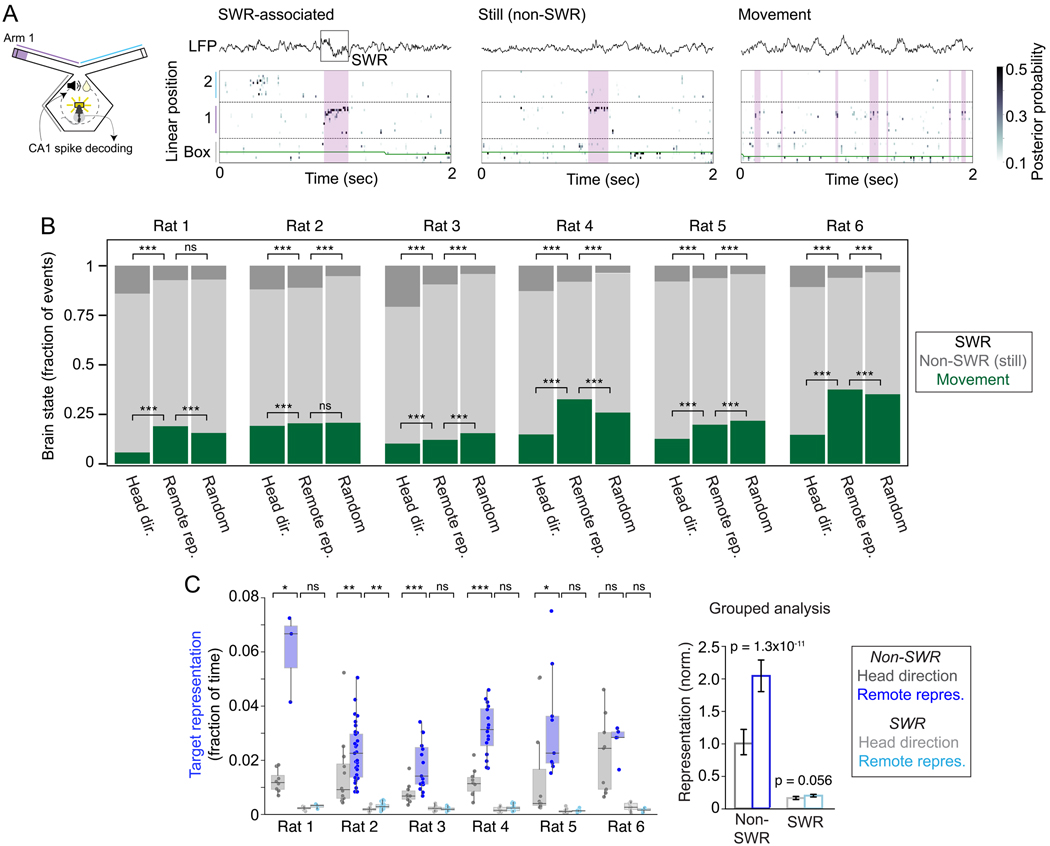
Brain state during remote representation. **(A)** Example remote representations during SWR, stillness outside of SWR (rat speed < 4cm/sec), and movement (rat speed > 4cm/sec). Left: schematic. Right: example plots; top: LFP trace, bottom: decoded linear position. **(B)** Summary of brain state during feedback periods. For each rat: remote representation (>40% posterior mass in target location) during head direction feedback, remote representation during neurofeedback, and random times. Chi-squared test and post-hoc z-test of proportions, ***: p<0.001. **(C)** Change in target representation during SWR-associated and non-SWR times for neurofeedback sessions compared to head direction feedback sessions. Similar analysis to Figure 4C. Grouped analysis (LME) for 6 rats. Mann-Whitney test (individual rats), *: p<0.05, **: p<0.01, ***: p<0.001. *n*: see Table S1. Box plots: center line is median, box is inner quartiles, whiskers are full distribution except outliers.

Previous work has identified remote spatial representations in the hippocampus in the context of two distinct physiological states. First, remote representations can be expressed during sharp-wave ripple (SWR) events. During waking, SWRs occur primarily during immobility, and spiking during SWRs is most strongly associated with memory consolidation processes^24,25,27,42^. Second, remote representations can be expressed in moving animals in the context of the ~8 Hz theta rhythm, where late phases of theta are most strongly associated with remote activity^21–23,43^. Remote activity during theta is hypothesized to contribute to memory retrieval in the context of deliberative decision making^23,44^. We therefore asked whether the remote spatial representations occurred during SWRs, theta, or in a different brain state^45^ (Fig. 7a).

Strikingly, remote representations were primarily expressed during stillness (head speed <4 cm/sec) but outside of clearly identifiable SWR events: only 6–11% of remote representations were associated with SWRs (Fig. 7b). Furthermore, target representations outside SWRs consistently increased in prevalence with neurofeedback (Fig. 7c, p < 0.05 in 5 of 6 individual rats, Mann-Whitney; grouped analysis: p = 1.3e-11, LME), while representations associated with SWRs did not consistently increase across animals (Fig. 7c, p < 0.05 in 1 of 6 rats, Mann-Whitney; grouped: p = 0.056, LME). Those remote representations that were expressed during movement showed characteristics similar to those seen in previous reports^3^. These representations were associated with specific theta phases in all rats (p < 0.0001 for each rat, Rayleigh test, Fig. S7a), and in 5 of 6 rats, remote representations were more prevalent in late theta phases.

We also compared the brain states during which remote representations were generated across the head direction and neurofeedback sessions. During neurofeedback, remote representations were more likely to occur during movement and less likely to occur during SWRs as compared to head direction feedback (Fig. 7b, Chi-square test and post-hoc Z-test of proportions for movement fraction and SWR fraction in each rat, p<0.001). This suggests that remote representations during neurofeedback occurred in more active and engaged states compared to those seen during head direction feedback. Within neurofeedback sessions, we compared brain states during remote representations to random times at the center reward port. Of note, SWRs were more frequent during times of remote representation compared to random times in 5 of 6 rats (Fig. 7b, Chi-square test and post-hoc Z-test of proportions for SWR fraction in each rat, p<0.001).

Given the surprising observation that most remote events occurred during stillness but outside of SWRs, we then asked whether there was a specific LFP signature associated with these events, as there is for immobility-related place activity^46^. We were not able to not identify such a signature, although some rats had increased power >100 Hz, potentially reflecting high gamma activity or spiking (Fig. S7b). Indeed, and as expected, multiunit spiking activity peaked at the time of remote representations (Fig. S7c). Finally, we also examined the relative prevalence, across brain states, of representations that jumped to the target location as compared to representations that included the intermediate locations along a trajectory from the animal to the target. We found both jump and trajectory representations across moving, still, and SWR periods with no consistent effects of prevalence across animals (Fig. S7d).

## DISCUSSION

We developed a closed-loop neurofeedback system for hippocampal representations and used this system to reward rats for generating specific remote spatial representations. Although no specific retrieval cues were presented, rats learned to activate specific representations corresponding to experimenter-chosen spatial locations. These representations typically jumped directly to the target region. Rats were also able to generate different representations at different times in response to changes in the target location, demonstrating flexibility. These remote representations engaged one or more cell assemblies and multiple neurons within each assembly, consistent with the reactivation of a population of neurons representing the remote location. Our work establishes a model for studying how the brain can deliberately reinstate representations related to previous experience in the absence of cues and specific behavioral outputs.

Studying memory retrieval as a distinct process requires distinguishing retrieval related activity from both the behaviors that retrieval can drive as well as the cues that can drive retrieval. Our work builds on previous brain-machine interface (BMI) approaches to make this possible. BMI systems can be used to drive animals to generate patterns of activity in the absence of associated behavior^47–49^, potentially enabling separation of retrieval from action. At the same time, previous approaches, including a recent demonstration of volitional control in the hippocampus^18^, use continuous sensory feedback to drive incremental changes in neural activity patterns that allow an animal to achieve a desired goal.

As our goal was to remove the potential confounds of pattern-specific sensory inputs, we instead required that animals remain near the center port of the maze (from which the experimenter-selected target region was not visible) and rewarded the animal for generating a representation of the target location. This approach had an additional critical benefit: it meant that animals could directly engage the representation of the target region without the requirement that they activate a series of intermediate representations. In the context of space, these would be the representations of the position between the animals’ actual location and the target presentation.

Our animals took advantage of that feature, generating representations that most often “jumped” to the target area without activating representations of intermediate locations. This enables study of a key aspect of memory: the ability to mentally teleport to places or events distant in space and time. The fact that the animals generated these distant events also argues strongly against a simple sensory explanation for their ability to activate the associated representations. From the neurofeedback location, the animal could see only the base of each of the two possible target arms. If visual cues were assisting the animal in generating a representation of the arm end (target), we would expect to see representation of the very beginning of the arm preceding representation of the target. However, we rarely observed these activity patterns immediately before target representations. Related to this, in the control experiment when the rat was rewarded for turning its head towards the target arm, the decoded representation remained in the box at the animal’s current location (Fig. S3d), further indicating that the remote representations generated during neurofeedback were not tied to the animal’s gaze. If cues in the visual field were driving remote representation, we would expect head turns towards the target to trigger remote representation as was recently reported in chickadees^50^.

Our work also provides insights into the brain states and activity patterns that can support the generation of remote representations consistent with memory retrieval. Most remote representations in this task occurred during stillness but did not overlap with SWRs, and remote representations that did occur during SWRs were not enriched despite many days of training. Thus, while it is possible to train animals to increase the rate of SWR generation^51^, these findings suggest that representations within SWRs are less amenable to control. This possibility is consistent with the idea that SWRs are critical for “offline” memory consolidation and updating functions rather than “online” retrieval to inform immediate decisions^25,24,27^.

We also found that some of the events occurred during movement, and consistent with previous observations^21,23,43^, these remote events were most often not in the trough of the theta rhythm where coding for the animal’s actual position is prevalent, but instead in the later phases of theta. This provides further support for the notion that specific theta phases are most conducive to the generation of non-local representations.

At the same time, most events were seen during periods of stillness and were not associated with an obvious local field potential signature. This state has also been associated with immobility-related place cell activity^46,52,53^, and our results indicate that in rats, the state is also consistent with a more “generative” function associated with remote representations^45^. Specifically, the lack of movement and associated sensory input may be conducive to the generation of internally driven patterns of activity associated with specific past experiences.

The specific mechanisms that support the generation of these remote representations are unknown, but previous studies of remote hippocampal representations in the context of SWRs^24,27^ and movement^21,28^ highlight the engagement of multiple cortical regions including the prefrontal cortex before, during, and after remote hippocampal events. It seems plausible that related networks are engaged when animals deliberately activate specific remote representations, but the mechanisms that might differentiate more automatic from more deliberate activation are not understood. More broadly, the generation of patterns of activity associated with past experience is central to the retrieval of memories^54^ and to related cognitive functions like planning and imagination^44^. The ability to train animals to generate such patterns in the absence of specific cues and specific behavioral outputs thereby provides a potentially powerful tool for understanding how the brain can support these remarkable functions.

### RESOURCE AVAILABILITY

#### Lead contact

Loren Frank, loren.frank@ucsf.edu

#### Materials Availability

Not applicable.

#### Data and Code Availability

The neural recording raw data is available on DANDI, with DOI: https://doi.org/10.48324/dandi.001280/0.241218.2300. Realtime decoding data is available on Dryad, with DOI: https://doi.org/10.5061/dryad.dz08kps72. Summarized results data files are available on Dryad, with DOI: https://doi.org/10.5061/dryad.dz08kps72. Jupyter notebook to generate all figures and analysis code is available on GitHub with DOI: https://doi.org/10.5281/zenodo.14522610. Real-time spatial decoder code is available on GitHub with DOI: https://doi.org/10.5281/zenodo.14399015 (current version) and DOI: https://doi.org/10.5281/zenodo.14399029 (version used in this manuscript). Any additional information required to reanalyze the data reported in this paper is available from the lead contact upon request.

## STAR METHODS

### Experimental models and subject details

Adult, male wild type Long Evans rats 6–9 months of age were obtained from Charles River Labs. Rats were housed at UCSF following all IACUC guidelines. All surgical procedures done following UCSF IACUC guidelines. Work was approved by UCSF IACUC.

### Methods details

#### Behavioral training

Rats were food restricted to 85% of their free body weight. Before neural implant surgery, rats were trained to nosepoke in reward ports to receive a milk reward on a linear track. Rats were then exposed to the task environment and trained in the task structure, in which they had to visit lit reward ports to receive a milk reward. The task environment consisted of a central area with a milk reward port and two attached arms with a reward port at each arm end. The task structure consisted of two phases during 40–45 minute recording sessions. In the first 10–15 minutes (exploration phase), the rat explored the Y-shaped environment (center box area, arm 1, and arm 2) and received milk reward by nosepoking in each lit reward port (300 uL of sweetened condensed milk was delivered automatically via the Trodes recording software). The light cues directed the rat to visit each arm 12 times and return to the center after each arm visit (arm 1 and arm 2 visits were randomly ordered). In the second phase (operant conditioning), lasting 20–30 minutes, the rat could only receive reward at the center port. During the conditioning phase, a sound cue was played and then a milk reward was available at the center port. In pretraining, the rat learned the association between the sound cue and reward: when the sound cue played, the rat had to nosepoke within 3 seconds to receive reward. During initial training the sound cue occurred randomly (centered on intervals of 5–30 seconds). For head direction feedback, the sound cue was triggered when the rat turned its head in a specific direction. For remote representation neurofeedback, the sound cue was triggered by online detection of a specific remote hippocampal representation (as in pretraining, the rat had to nosepoke within 3 seconds of the sound cue to receive the milk reward).

#### Neural implant and surgery

Rats that completed the pretraining were surgically implanted with a microdrive containing 64 individually moveable tetrodes targeting dorsal hippocampus region CA1^55^. The microdrive was assembled from 3D printed plastic shuttles and drive body. Individual tetrodes bundles (nichrome wire, bundled, cut sharply, and gold plated) were attached to each shuttle and threaded through the drive body to exit via two cannulas. An implant surgery^25^ was performed in compliance with the UCSF IACUC animal safely protocols to attach the microdrive to the animal’s skull. During surgery, bilaterial craniotomies and durotomies were made for the two cannulas (A/P: 4 mm behind Bregma, M/L: +/− 2.6 mm around the sagittal suture). The microdrive containing the tetrodes and shuttles was attached to the skull with dental cement and steel skull screws. A skull screw above the cerebellum served as an electrical reference for the recordings. Following surgery, tetrodes were lowered to the dorsal CA1 pyramidal cell layer over 2–3 weeks.

#### Data collection

Hippocampal tetrode recordings were collected using SpikeGadgets hardware and Trodes recording software (sampling rate, 30 kHz). The rat’s physical position was recorded using a Manta camera (frame rate 30 Hz) and red and green LEDs attached to the headstage were tracked using Trodes. These LEDs allowed calculation of head direction.

#### Head direction feedback

After surgery recovery and tetrode adjusting, each rat started with 4 days of head direction conditioning. Each training day included 3 recording sessions. The recording sessions followed the task structure described above. During the feedback part of the task, the sound cue and reward was triggered by the rat’s head direction. Reward cue was triggered by a head turn to either 30 degrees to left (arm 1) or to the right (arm 2) when facing forward towards the base of the outer arms. The angular accuracy required to trigger the reward cue increased over the first 25 rewards of each feedback session (from +/− 20 degrees to +/− 3 degrees). The feedback phase lasted for 30 minutes or until the rat received 75 rewards, whichever occurred first. For the first 2 days, the target direction was pointing towards one arm and then the target switched to the opposite arm for the next 2 days.

#### Remote representation neurofeedback

Following head direction feedback, the rat switched to remote representation neurofeedback. Each day had 3 recording sessions and the target location (either the distal 25 cm of arm 1 or arm 2) switched every 3 days. Based on recording quality rats were trained for 6 days (rat 1) or 18 days (rats 2–6). During the exploration phase at the beginning of each session, the recorded CA1 spikes were used to build an encoding model that associated spiking activity with spatial locations in the environment. Then, during the feedback phase, when the decoder (described in more detail below) detected a coherent representation of the target location, the sound cue was triggered and reward was available at the center port. The following requirements had to be met to trigger the reward cue: 30 msec running average of decoded position, >40% in target arm end, <20% in opposite arm, <20% in box area, at least 2 tetrodes with spatially specific spikes during 30 msec window, and rat physical distance to center port < 17cm. The feedback phase lasted for 30 minutes or until the rat received 75 rewards, whichever occurred first. In rat 1, only 1 tetrode with spatially specific spikes was required.

#### Histology

Rat brains were fixed with 4% PFA, serially sectioned, and then treated with Nissl staining to visualize hippocampal cellular structure and electrolytic lesions (made after the experiment) at the tetrode recording sites.

### Quantification and statistical information

#### Data processing

Raw electrical signal, position tracking, and reward port nosepokes were extracted from the Trodes recording file and saved as an NWB file (https://github.com/orgs/LorenFrankLab/rec_to_nwb). The NWB file was inserted into a database organized by the Spyglass package for reproducible analysis of neural data (https://github.com/orgs/LorenFrankLab/spyglass)^56^. All subsequent analysis was done on data within the database.

#### Clusterless decoding of hippocampal representation

Once tetrodes were lowered to the pyramidal cell layer, hippocampal representations were decoded from these recordings using a spatial decoding algorithm. To decode position from action potential firing (spiking) of CA1 neurons, this algorithm builds a marked point-process model to relate all CA1 spikes (above a set threshold) to the rat’s physical position. Each mark in this model has five dimensions (4 voltages, from each tetrode wire, and the rat’s physical position (linearized) in the environment). To decode the hippocampal spatial representation (mental position), the 4 voltage dimensions of each new spike are compared to the model and the estimated (mental) position is calculated by a weighted sum of the positions corresponding to the spikes in the encoding model (closest marks to the new mark have highest weight). Position is estimated for all spikes in a small time window (6 msec) and these estimates are combined (by taking the product) to generate a prediction of the spatial position represented by the combined hippocampal activity during each time window. A key feature of this decoding algorithm is that it does not require spike sorting (“clusterless”).^35^

#### Real-time spatial decoding

The clusterless decoding algorithm was implemented for fast operation using a desktop computer with 28 cores and parallelization of the decoding algorithm using message passing interface (MPI). During each recording session, the system decoded ~1 million spikes. With a decoding latency of 30 msec, 90–99% of incoming spikes were decoded. The details of the real-time decoder are described in an accompanying manuscript^32^.

#### Real-time decoder output

The decoded mental position as estimated by the real-time decoder was saved each session in a separate recording file. This file included the rat’s actual position and decoded position for every 6 msec time bin and each detected reward event (head direction or remote representation).

#### Data inclusion

Recording sessions were included with high quality spatial decoding. High quality sessions were defined as: during the exploration period, when the rat was running outward from the box and in the end of the target arm, the posterior maximum of the decoded position was in the target arm in at least 65% of time bins.

A few analyses could not be performed on all recording sessions because of errors with data saving and preprocessing. The missing sessions are listed here.

- Figures 1–5: no missing data.
- Figure 6: rat 1 (2 missing sessions), rat 2 (2 missing), rat 3 (1 missing), rat 4 (1 missing), rat 5 (4 missing), rat 6 (3 missing).
- Figure 7: rat 1 (4 missing head direction, 2 missing neurofeedback), rat 2 (0 missing), rat 3 (1 missing neurofeedback), rat 4 (0 missing), rat 5 (4 missing neurofeedback), rat 6 (3 missing head direction, 2 missing neurofeedback).

#### Spike Sorting

The raw tetrode recording was band-pass filtered from 600–6000 Hz and spikes were detected and sorted using MountainSort4^40^. During subsequent curation, noise clusters and multiunit clusters were removed automatically. Manual inspection was used to merge clusters that were inappropriately split. The remaining clusters are considered “isolated single units.”

#### LFP analysis

For LFP analysis, the raw tetrode recordings were band-pass filtered from 0 – 400 Hz. Previously described methods were used to detect sharp wave ripples^25^. Theta phase was calculated from one tetrode in each rat located above the CA1 pyramidal cell layer (stratum oriens).

#### Cell assembly analysis

Cell assemblies were identified using combined PCA and ICA with methods described previously^39^. The spike times of all isolated single units for a single recording session were binned into 30 msec bins and then co-firing cell assemblies were detected.

## Supplementary Material

1

## Figures and Tables

**Table:** Key resources table

REAGENT or RESOURCE	SOURCE	IDENTIFIER
Antibodies		
		
		
		
		
		
Bacterial and virus strains		
		
		
		
		
		
Biological samples		
		
		
		
		
		
Chemicals, peptides, and recombinant proteins		
		
		
		
		
		
Critical commercial assays		
		
		
		
		
		
Deposited data		
Raw electrophysiology data	https://doi.org/10.48324/dandi.001280/0.241218.2300	
Real-time decoder data	https://doi.org/10.5061/dryad.dz08kps72	
		
		
		
Experimental models: Cell lines		
		
		
		
		
		
Experimental models: Organisms/strains		
Adult, male, wild type Long Evans rats.	Charles River	Cat#: Crl:LE 006
		
		
		
		
		
Oligonucleotides		
		
		
		
		
		
Recombinant DNA		
		
		
		
		
		
Software and algorithms		
Trodes neural acquisition software.	SpikeGadgets	
Real-time decoding algorithm (current version)	Chu et al., 2024^32^ https://doi.org/10.5281/zenodo.14399015	
Real-time decoding algorithm (this manuscript)	https://doi.org/10.5281/zenodo.14399029	
Spyglass	https://github.com/LorenFrankLab	
Trodes-to-nwb	https://github.com/LorenFrankLab	
Custom analysis scripts for this manuscript	https://doi.org/10.5281/zenodo.14522610	
Other		
Data acquisition system	SpikeGadgets	
Nichrome tetrode wire, 0.0005 inches diameter	Alleima	Cat#: PX000029
		
		
		

## Bullet points:

Highlights are 3–4 bullet points of no more than 85 characters in length, including spaces, and they summarize the core results of the paper in order to allow readers to quickly gain an understanding of the main take-home messages of the paper.

### Bullet points:

- Real-time neurofeedback to promote spatial representations of remote locations
- With neurofeedback, rats can generate specific remote representations
- Remote representations often jump directly to distant, not-visible locations
- Remote representations primarily occur outside sharp wave ripples
