# Supplementary material for: Closed-loop modulation of remote hippocampal representations with neurofeedback
Source: Neuron. Author manuscript; Available in PMC 2025 May 12. (PMC12067296; doi:10.1016/j.neuron.2024.12.023)
Supplement: 1 [file NIHMS2048106-supplement-1.pdf]

Supplemental Figures.

Closed-loop modulation of remote hippocampal representations with neurofeedback

Michael E. Coulter, Anna K. Gillespie, Joshua Chu, Eric L. Denovellis, Trevor T.K. Nguyen, Daniel F. Liu, Katherine Wadhwani, Baibhav Sharma, Kevin Wang, Xinyi Deng, Uri T. Eden, Caleb Kemere, Loren M. Frank

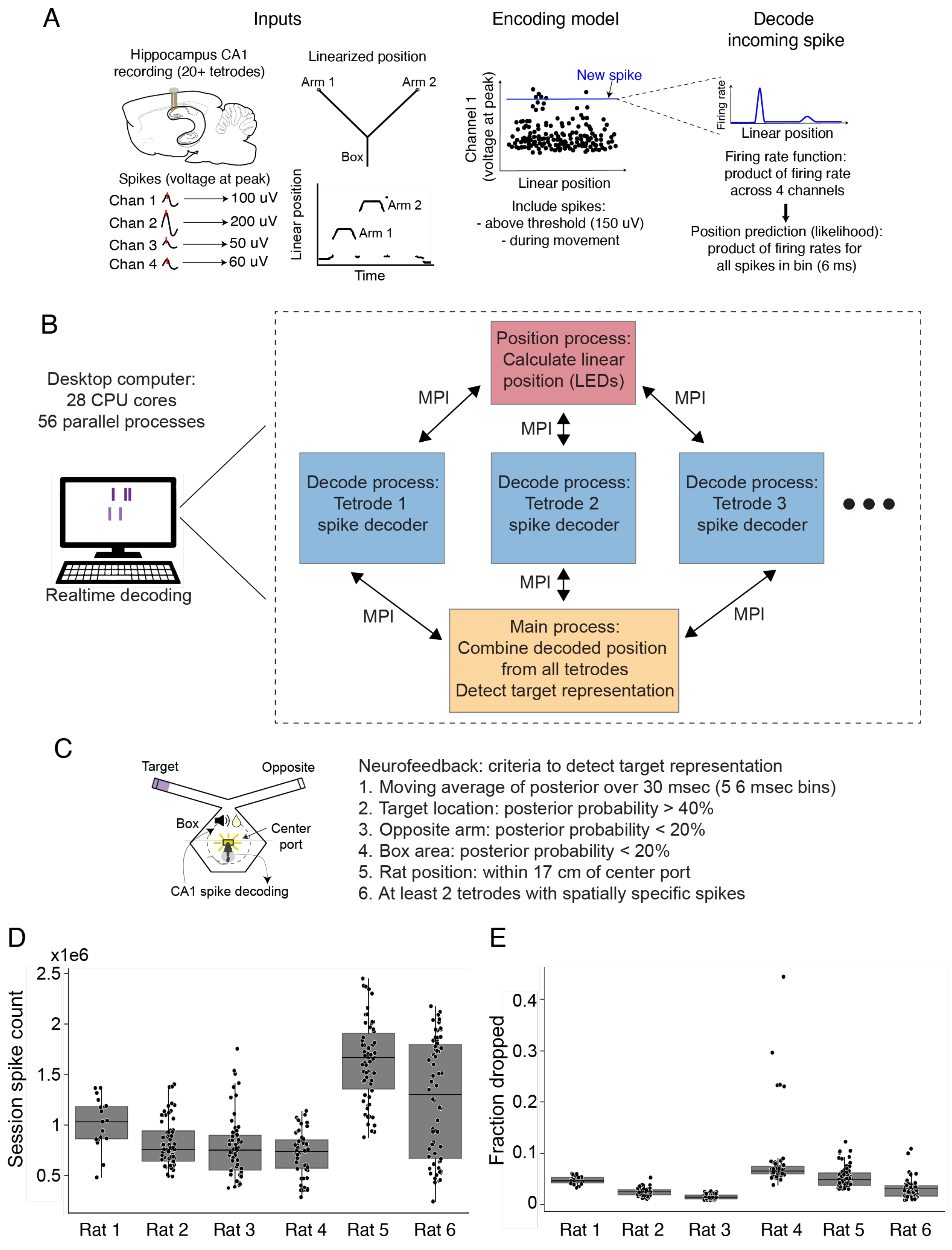
A
Inputs
Hippocampus CA1
recording (20+ tetrodes)
Spikes (voltage at peak)
Chan 1 → 100 uV
Chan 2 → 200 uV
Chan 3 → 50 uV
Chan 4 → 60 uV
Linearized position
Arm 1
Arm 2
Box
Linear position
Time
Encoding model
New spike
Channel 1 (voltage at peak)
Linear position
Include spikes:
- above threshold (150 uV)
- during movement
Decode incoming spike
Firing rate
Linear position
Firing rate function: product of firing rate across 4 channels
Position prediction (likelihood): product of firing rates for all spikes in bin (6 ms)
B
Desktop computer:
28 CPU cores
56 parallel processes
Realtime decoding
Position process: Calculate linear position (LEDs)
MPI
Decode process: Tetrode 1 spike decoder
Decode process: Tetrode 2 spike decoder
Decode process: Tetrode 3 spike decoder
Main process: Combine decoded position from all tetrodes
Detect target representation
C
Target
Opposite
Box
Center port
CA1 spike decoding
Neurofeedback: criteria to detect target representation
1. Moving average of posterior over 30 msec (5 6 msec bins)
2. Target location: posterior probability > 40%
3. Opposite arm: posterior probability < 20%
4. Box area: posterior probability < 20%
5. Rat position: within 17 cm of center port
6. At least 2 tetrodes with spatially specific spikes
D
x1e6
Session spike count
2.5
2
1.5
1
0.5
Rat 1
Rat 2
Rat 3
Rat 4
Rat 5
Rat 6
E
Fraction dropped
0.4
0.3
0.2
0.1
0
Rat 1
Rat 2
Rat 3
Rat 4
Rat 5
Rat 6

Figure S1: Real-time decoding algorithm and implementation, related to Figure 1. (A) Schematic of clusterless decoding algorithm. **(B)** Software architecture for real-time decoder implementation. MPI: message passing interface. **(C)** Criteria for detection of remote representation to trigger reward cue. **(D)** Total recording session spike counts for each rat. *n*: Rat 1: n=19 sessions, Rat 2: n=62. Rat 3: n=53. Rat 4: n=55. Rat 5: n=62. Rat 6: n=63. **(E)** Fraction dropped spikes for each rat (not decoded because of spike latency > 30 msec). *n*: Rat 1: n=19 sessions, Rat 2: n=62. Rat 3: n=53. Rat 4: n=55. Rat 5: n=62. Rat 6: n=63.

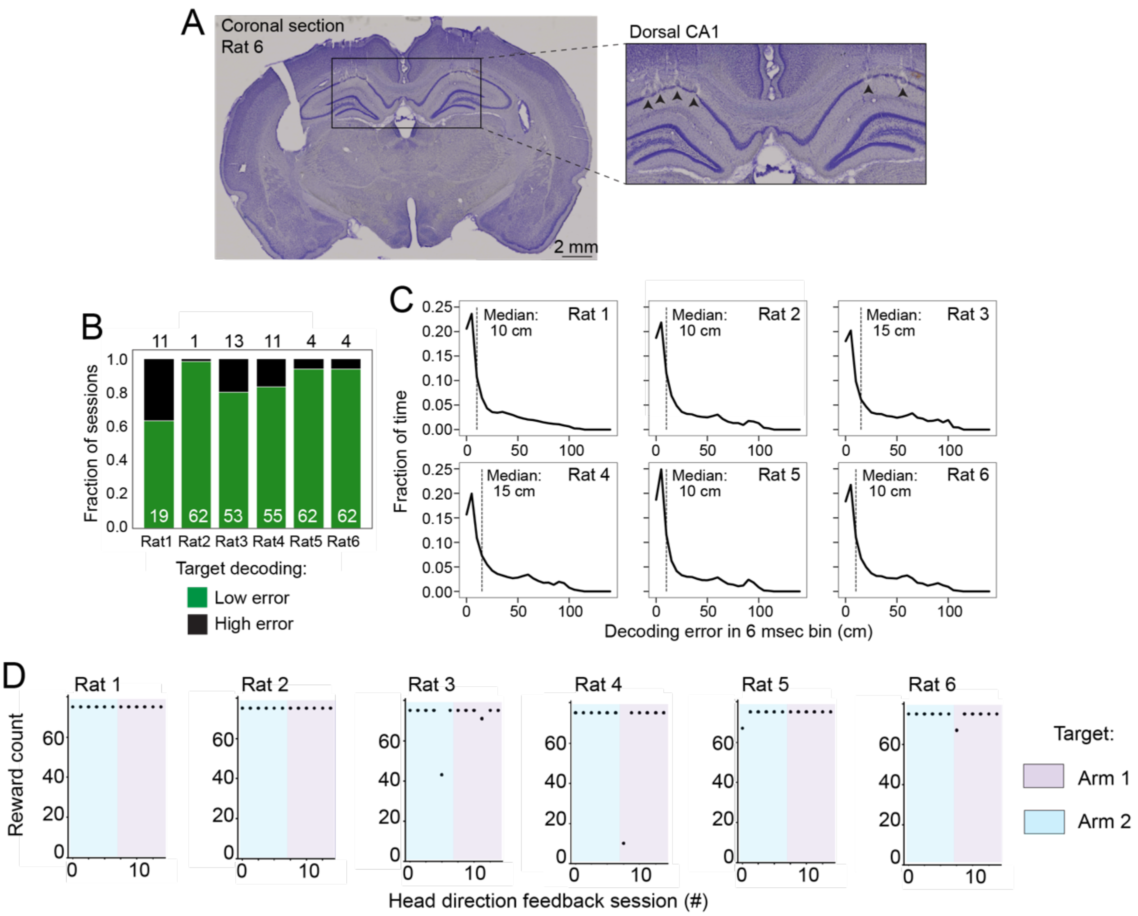

Figure S2: Tetrode placement, decoder performance, and head direction feedback, related to Figures 1 and 2. (A) Histology (Nissl stain) showing recording tetrodes located in dorsal CA1 of hippocampus. **(B)** Fraction of recording sessions for each rat with low decoding error vs. high decoding error (low: <35% decoding error in target region). **(C)** Decoding error during movement. Histogram of error (distance between rat's real position and decoded mental position) for each 6 msec time bin while the rat was moving (>4cm/sec). Median is marked with dashed vertical line. *n*: Rat 1: n=19 sessions, Rat 2: n=62. Rat 3: n=53. Rat 4: n=55. Rat 5: n=62. Rat 6: n=63. **(D)** Reward counts for all head direction feedback sessions.

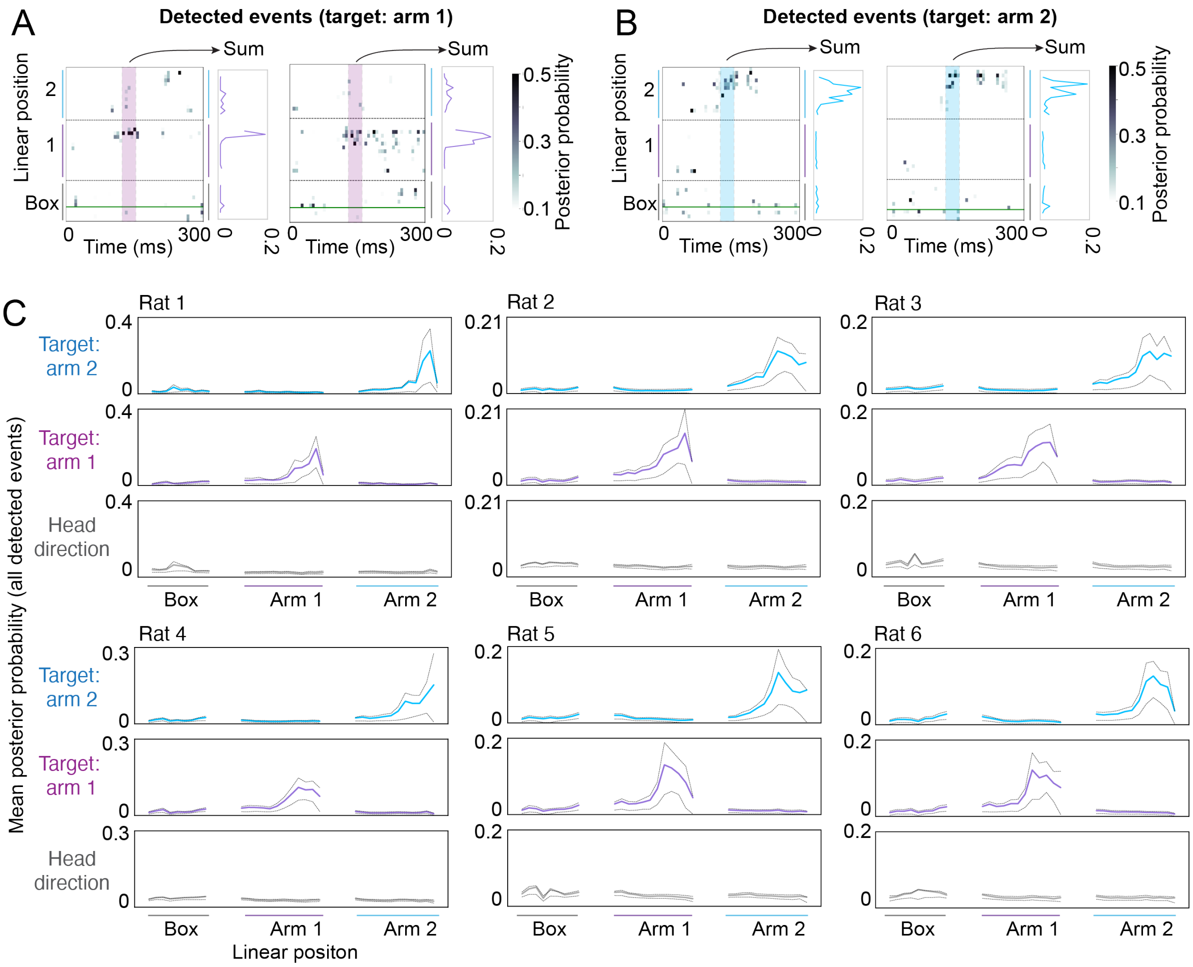

Figure S3: Summary of detected remote representations, related to Figure 3. (A) The rats received reward when the decoded position matched the target location. Examples of rewarded representations for arm 1 (**A**) and arm 2 (**B**). Shaded areas contain time bins that contribute to detection (30 msec). The linearized sum of the representation is shown for each example (right plot). **(C)** Hippocampal representation averaged across all detected events. Organized by target region: arm 2 neurofeedback (blue), arm 1 neurofeedback (purple), or head direction feedback (grey). Posterior probability was averaged over 30 msec that triggered detection (as show in **(A)** and **(B)**). Dashed lines represent 75% confidence interval.

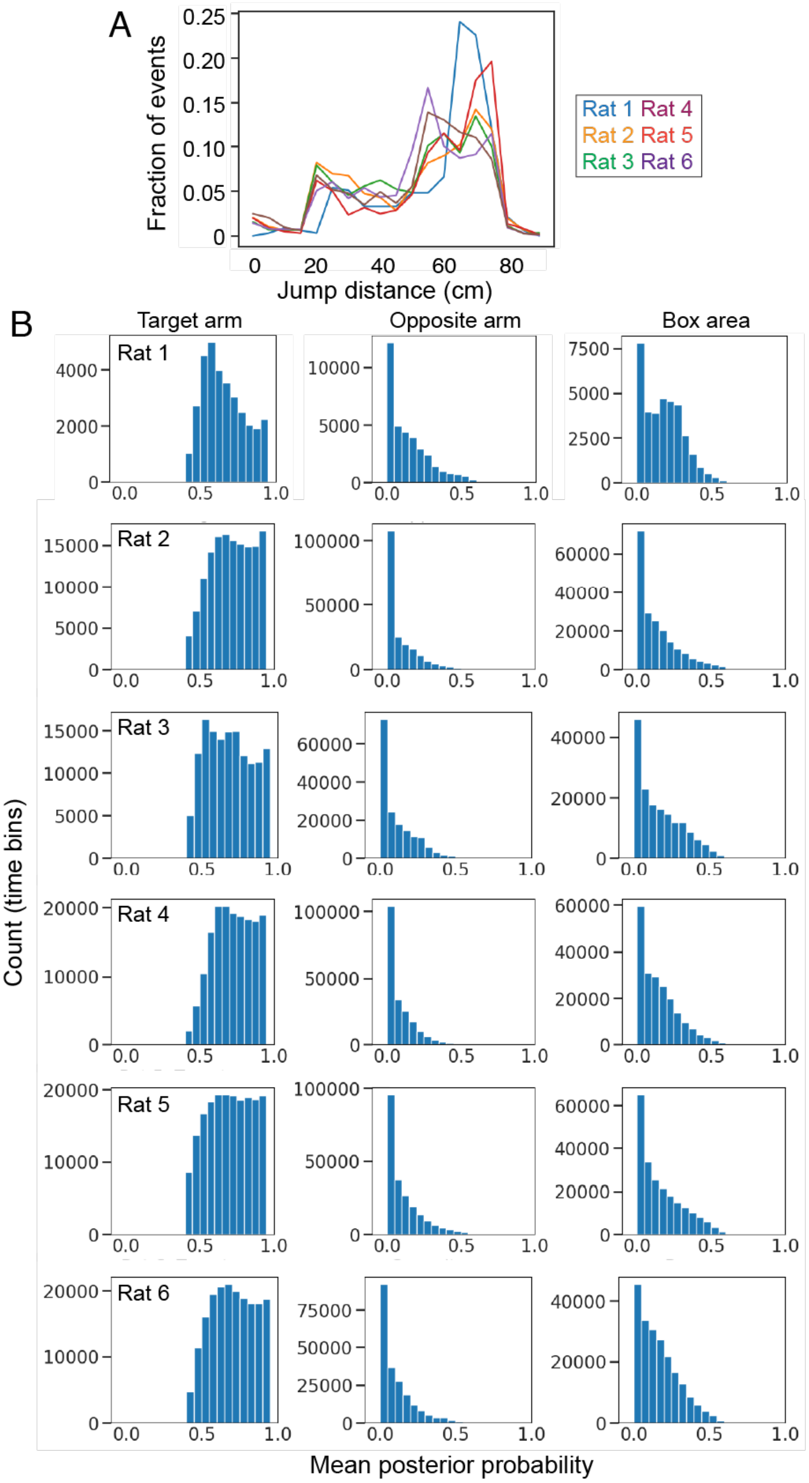

Figure S4. Additional quantifications of remote representations, related to Figures 3 and 4. (A) Distance between rat's position and nearest high-confidence representation in target arm for detected events (posterior mass > 0.4). Includes 90 msec before detection. **(B)** Decoded posterior probability at target representation times (>40% of posterior mass in target location). This shows that for each rat, nearly all times of target representation had at least 50% representation in the target arm and much less representation of either the opposite arm or the local area (box).

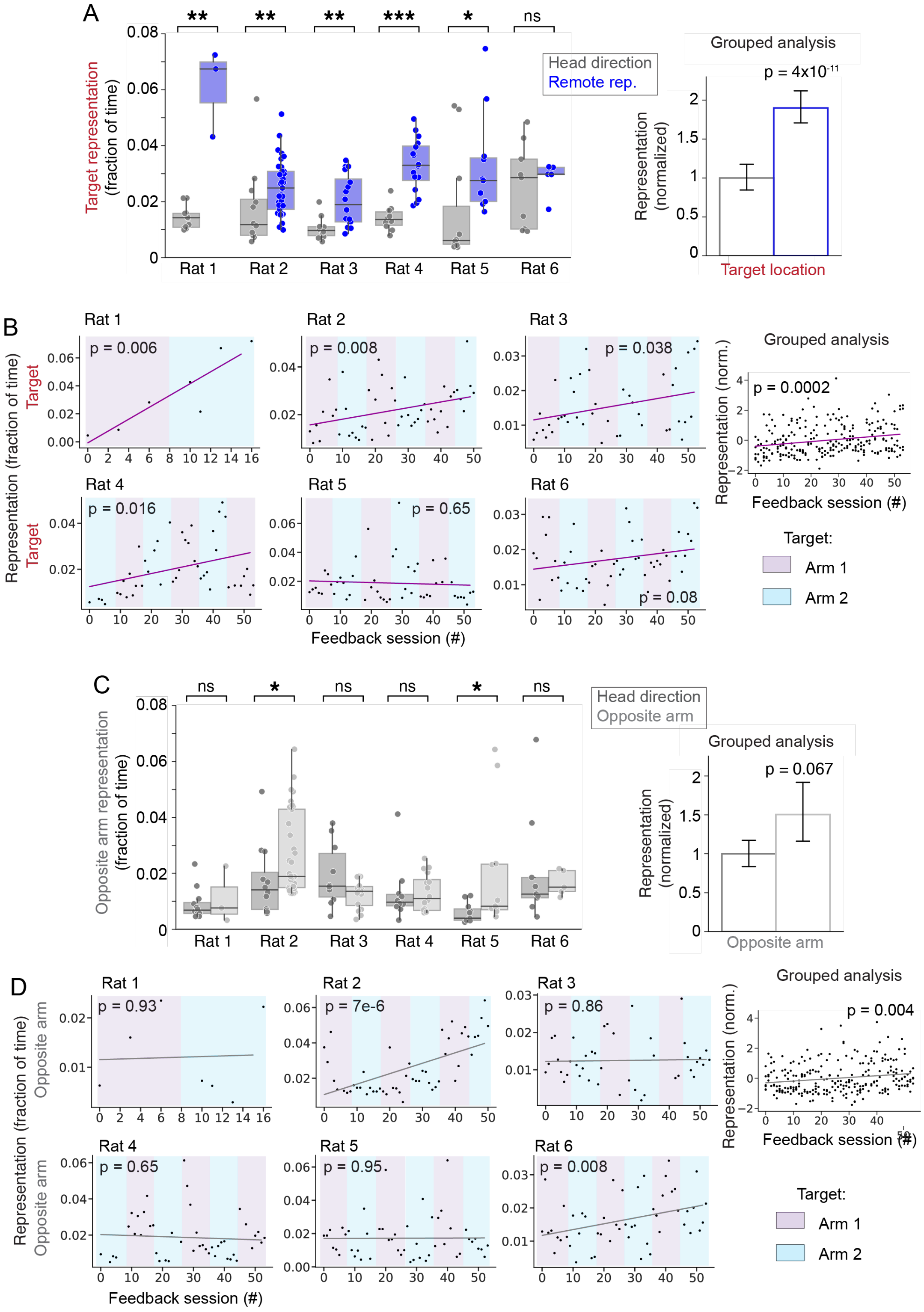

Remote representations with trigger-event removed and of opposite arm, related to Figures 4 and 5. (A) Same plots as in Figure 4C with triggering representation removed. *n*: Rat 1: Head direction: n=12 sessions, neurofeedback: n=3. Rat 2: Head direction: n=12, neurofeedback: n=33. Rat 3: Head direction: n=10, neurofeedback: n=16. Rat 4: Head direction: n=10, neurofeedback:

n=16. Rat 5: Head direction: n=11, neurofeedback: n=11. Rat 6: Head direction: n=9, neurofeedback: n=5**.** **(B)** Same plots as in Figure 5B with triggering representation removed. *n*: Rat 1: n=7 sessions, Rat 2: n=50. Rat 3: n=42. Rat 4: n=44. Rat 5: n=50. Rat 6: n=51. Mann-Whitney test, *: p<0.05, **: p<0.01, ***: p<0.001. **(C)** Same plots as in Figure 4C showing representation of opposite arm end (non-target). *n*: Rat 1: Head direction: n=12 sessions, neurofeedback: n=3. Rat 2: Head direction: n=12, neurofeedback: n=33. Rat 3: Head direction: n=10, neurofeedback: n=16. Rat 4: Head direction: n=10, neurofeedback: n=16. Rat 5: Head direction: n=11, neurofeedback: n=11. Rat 6: Head direction: n=9, neurofeedback: n=5. **(D)** Same plots as in Figure 5B showing representation of opposite arm end. *n*: Rat 1: n=7 sessions, Rat 2: n=50. Rat 3: n=42. Rat 4: n=44. Rat 5: n=50. Rat 6: n=51. Mann-Whitney test, *: p<0.05.

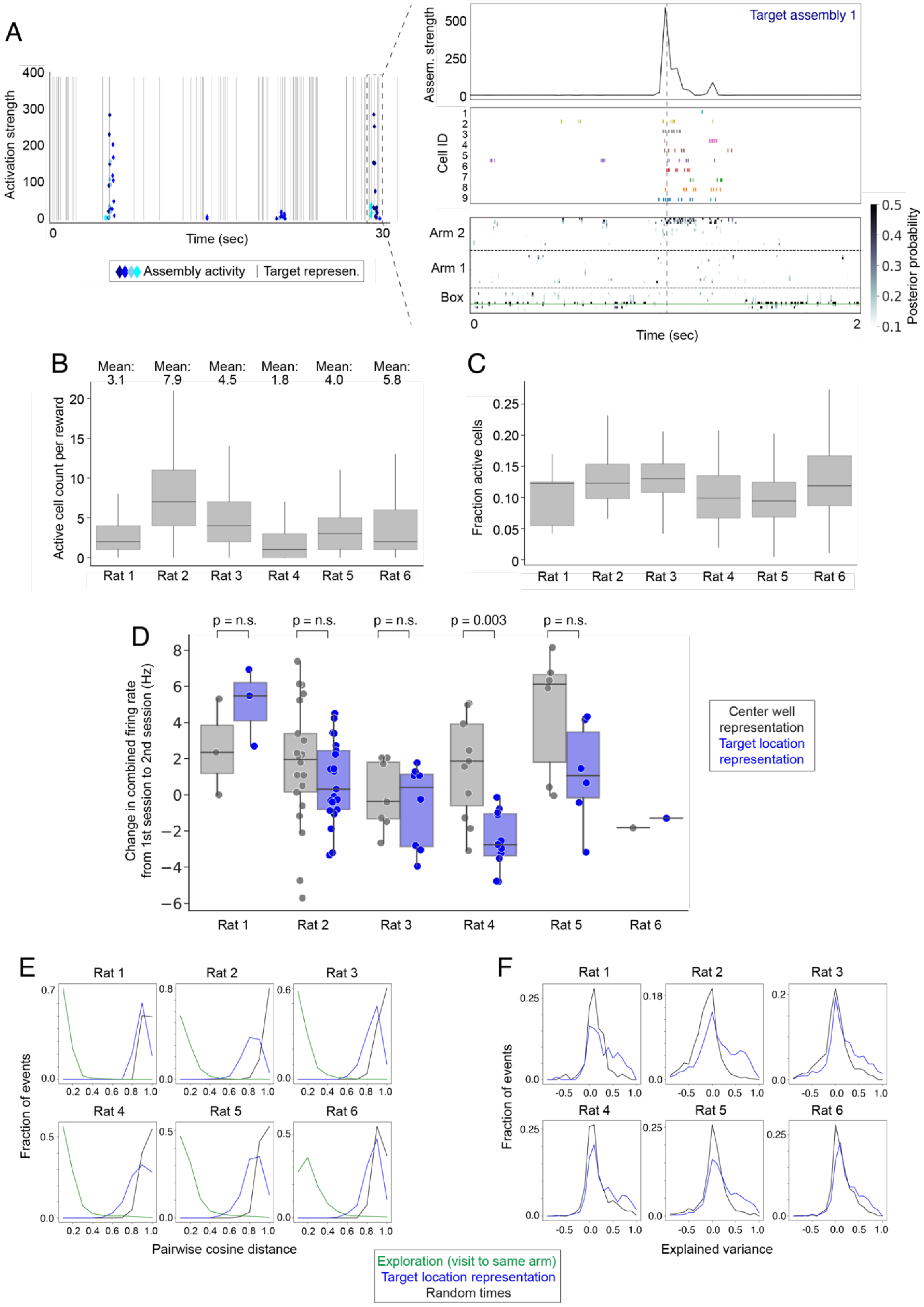
A
Activation strength
Time (sec)
Assembly activity
Target represen.
Reward cue
Target assembly 1
Assem. strength
Cell ID
Arm 2
Arm 1
Box
Time (sec)
Posterior probability
B
Mean: 3.1
Mean: 7.9
Mean: 4.5
Mean: 1.8
Mean: 4.0
Mean: 5.8
Active cell count per reward
Rat 1
Rat 2
Rat 3
Rat 4
Rat 5
Rat 6
C
Fraction active cells
D
p = n.s.
p = n.s.
p = n.s.
p = 0.003
p = n.s.
Change in combined firing rate from 1st session to 2nd session (Hz)
Center well representation
Target location representation
E
Fraction of events
Pairwise cosine distance
F
Explained variance
Exploration (visit to same arm)
Target location representation
Random times

Figure S6: Additional analyses of cell assembly activity, related to Figure 6. (A) Left: same panel from Figure 3F, showing target assembly activity (blue diamonds) and remote representation (grey lines). Right: zoom in on a detected target representation. During this representation, target assembly activity is high (top) and there are spikes from several high-strength cells within this assembly during the representation (middle). Clusterless decoding showing the target representation at the end of arm 2 (bottom). **(B)** We counted the number of high-strength cells (>5 s.d. above mean) with spiking activity immediately before target representations and found that, on average, all rats have more than one spiking cell (within 100 ms). *n*: Rat 1: n=322 reward times, Rat 2: n=2999. Rat 3: n=1970. Rat 4: n=1737. Rat 5: n=2037. Rat 6: n=1176. **(C)** This plot shows the fraction of high-strength cells active before each target representation. The fraction is similar across rats showing that much of the variability in cell count in **(B)** is the result of variations in the number of high-strength cells across rats, an indicator of recording quality. *n*: Rat 1: n=322 reward times, Rat 2: n=2999. Rat 3: n=1970. Rat 4: n=1737. Rat 5: n=2037. Rat 6: n=1176. **(D)** Population-level remapping analysis. Following high reward sessions (>90% possible rewards), change in representation of neurofeedback location at center reward port (gray) and target region (blue) are shown for the subsequent session. There was no consistent pattern of change observed across animals (2 rats center port > target, 3 rats target > center port). Rat 4 had a significantly larger increase in representation for the center port compared to the target region (Mann-Whitney, p=0.003). **(E)** Cosine distances between population vectors of combined single neuron spiking (cells: > 50 spikes and average firing rate < 7 Hz, spiking duration: 50 msec). Distance between population vectors was calculated for each of the following pairwise comparisons: exploration arm visit vs. exploration arm visit, exploration arm visit vs. remote representation, exploration arm visit vs. random time. Distance between vectors was smallest (most similar) for arm visit vs. arm visit, then arm visit vs. remote representation, and largest for arm visit vs. random time. For each rat, distance was significantly different for remote representations vs. random times (p < 0.0001 Mann-Whitney test). **(F)** A PCA model was built using single neuron spiking during exploration of the target location. Each visit (12) to the location was an item in the model. Then spiking activity during reward-triggering remote representations or random times (50 msec each) was fit with this model. Plot: explained variance for actual remote representation spiking activity compared to remote representation spiking predicted by the PCA model. For each rat, explained variance was higher for remote representations than for random times during the neurofeedback period (p < 0.0001 Mann-Whitney test).

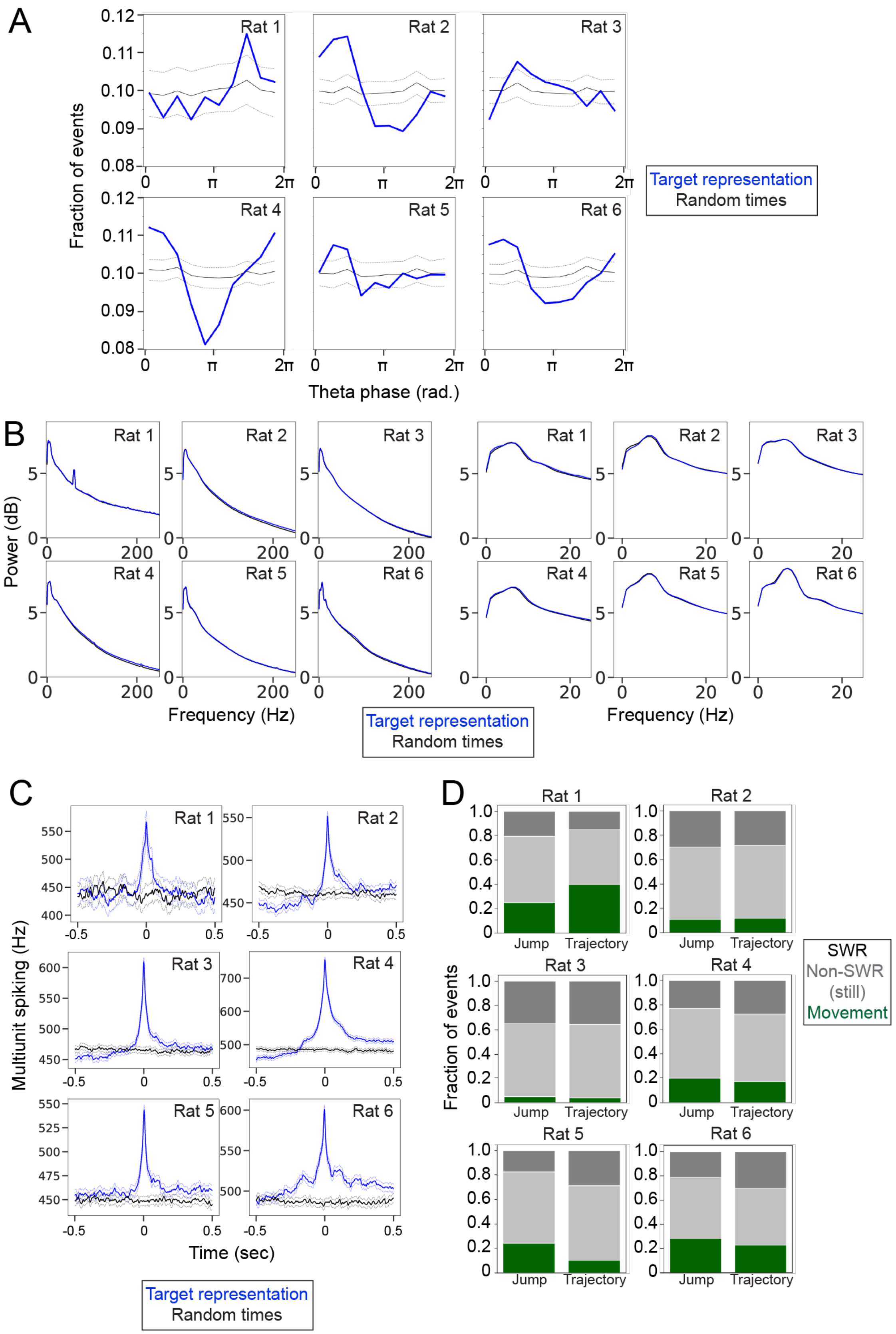
A
Fraction of events
Rat 1
Rat 2
Rat 3
Rat 4
Rat 5
Rat 6
Theta phase (rad.)
Target representation
Random times
B
Power (dB)
Frequency (Hz)
Target representation
Random times
C
Multiunit spiking (Hz)
Time (sec)
Target representation
Random times
D
Fraction of events
Jump
Trajectory
SWR
Non-SWR (still)
Movement

Figure S7: Additional analyses of brain state during remote representations, related to Figure 7. (A) Remote representation times (blue) during movement times are modulated by theta phase. Dashed lines are 95% confidence interval for theta phase of random times. All possible theta phase values were grouped into 10 equal bins and the fraction of remote representation events in each bin is plotted. To create null distribution (grey line with 95% confidence interval dashed lines) theta phase was calculated at random time bins matching the number of remote representation time bins. The random sampling was done 1000 times and the distribution was used to calculate the 95% confidence interval. *n*: Rat 1: n=13822 representation times. Rat 2: n=42027. Rat 3: n=21747. Rat 4: n=76860. Rat 5: n=37556. Rat 6: n=71226. **(B)** LFP spectrogram of remote representation times (1 sec surrounding remote representation). Left: tetrode in CA1 cell layer (referenced), right: tetrode above cell layer (unreferenced). Theta power is most accurately measured above the cell layer with an unreferenced tetrode, and so right plot is zoomed in to low frequency range. *n*: Rat 1: n=1071 representation times (1 sec non-overlapping bins, rat still, non-SWR). Rat 2: n=16032. Rat 3: n=13605. Rat 4: n=17164. Rat 5: n=15025. Rat 6: n=14241. **(C)** Multiunit spiking at times of remote representation (1 sec surrounding representation). Blue: remote representation, black: random times. Dashed lines: 95% confidence intervals of the mean. *n*: Rat 1: n=1840 representation times (1 sec non-overlapping bins). Rat 2: n=18211. Rat 3: n=22150. Rat 4: n=21021. Rat 5: n=23581. Rat 6: n=23092. **(D)** Brain state associated with different types of remote representations (either jump or trajectory representations). There were no consistent differences across animals in the brain state associated with jump (remote representation confined to target location with or without center port) or trajectory (posterior with significant linear regression covering at least 35 cm in target arm) remote representations.

Figure 4c	*n*: Rat 1: Head direction: n=12 sessions, neurofeedback: n=3. Rat 2: Head direction: n=12, neurofeedback: n=33. Rat 3: Head direction: n=10, neurofeedback: n=16. Rat 4: Head direction: n=10, neurofeedback: n=16. Rat 5: Head direction: n=11, neurofeedback: n=11. Rat 6: Head direction: n=9, neurofeedback: n=5.<br>Mann-Whitney test: Rat 1: p=0.0044. Rat 2: p=0.0049. Rat 3: p=0.0014. Rat 4: p=5.5e-5. Rat 5: p=0.015. Rat 6: p=0.52.
Figure 4d	*n*: same as Figure 4c.<br>Mann-Whitney test: Rat 1: p=0.23. Rat 2: p=0.43. Rat 3: p=0.77. Rat 4: p=0.30. Rat 5: p=0.32. Rat 6: p=0.80.
Figures 5b and 5c	*n*: Rat 1: n=7 sessions, Rat 2: n=50. Rat 3: n=42. Rat 4: n=44. Rat 5: n=50. Rat 6: n=51.<br>Linear regression: labeled on figure.
Figure 6e	*n*: Rat 1: target: n=8 cell assemblies, random: n=8, non-target: n=72. Rat 2: target: n=126, random: n=126, non-target: n=694. Rat 3: target: n=81, random: n=81, non-target: n=527. Rat 4: target: n=57, random: n=57, non-target: n=568. Rat 5: target: n=85, random: n=85, non-target: n=578. Rat 6: target: n=53, random: n=53, non-target: n=769.<br>Mann-Whitney test: Rat 1: target v. random: p=1.6e-4, target v. non-target: p=2.8e-7. Rat 2: target v. random: p<1e-10, target v. non-target: p<1e-10. Rat 3: target v. random: p<1e-10, target v. non-target: p<1e-10. Rat 4: target v. random: p<1e-10, target v. non-target: p<1e-10. Rat 5: target v. random: p<1e-10, target v. non-target: p<1e-10. Rat 6: target v. random: p<1e-10, target v. non-target: p<1e-10.
Figure 6g	*n*: Rat 1: target: n=8 cell assemblies, random: n=8, non-target: n=72. Rat 2: target: n=126, random: n=126, non-target: n=694. Rat 3: target: n=81, random: n=81, non-target: n=527. Rat 4: target: n=57, random: n=57, non-target: n=568. Rat 5: target: n=85, random: n=85, non-target: n=578. Rat 6: target: n=53, random: n=53, non-target: n=769.<br>Mann-Whitney test: Rat 1: target v. random: p=0.16, target v. non-target: p=4.7e-3. Rat 2: target v. random: p<1e-10, target v. non-target: p<1e-10. Rat 3: target v. random: p<1e-10, target v. non-target: p=6.4e-9. Rat 4: target v. random: p<1e-10, target v. non-target: p=0.45. Rat 5: target v. random: p<1e-10, target v. non-target: p=1.7e-6. Rat 6: target v. random: p=8.4e-6, target v. non-target: p=0.40.
Figure 7c	*n*: Rat 1: Head direction: n=8 sessions, neurofeedback: n=3. Rat 2: Head direction: n=12, neurofeedback: n=33. Rat 3: Head direction: n=10, neurofeedback: n=15. Rat 4: Head direction: n=10, neurofeedback: n=16. Rat 5: Head direction: n=11, neurofeedback: n=9. Rat 6: Head direction: n=9, neurofeedback: n=5.<br>Non-SWR:<br>Mann-Whitney test: Rat 1: p=0.012. Rat 2: p=0.0079. Rat 3: p=0.00079. Rat 4: p=5.5e-5. Rat 5: p=0.023. Rat 6: p=0.36.<br>SWR:<br>Mann-Whitney test: Rat 1: p=0.28. Rat 2: p=0.0011. Rat 3: p=0.45. Rat 4: p=0.26. Rat 5: p=0.22. Rat 6: p=0.15.

Table S1: Number of samples and statistical tests for main figures, related to Figures 4,5,6,7.
